# A case of scrofuloderma of the axilla presenting as hidradenitis suppurativa: A case report

**DOI:** 10.1177/2050313X221117706

**Published:** 2022-10-07

**Authors:** Laurence Garon, Ashley Hill

**Affiliations:** Division of Dermatology, Department of Medicine, Centre Hospitalier de l’Université de Montréal (CHUM), Montréal, QC, Canada H2X 0C1

**Keywords:** dermatology, infectious disease

## Abstract

Scrofuloderma is an uncommon cutaneous presentation of tuberculosis. It can be difficult to diagnose, as it can mimic various skin conditions, including hidradenitis suppurativa. We report a case of a 46-year-old female refugee patient with a history of nodules and sinus tracts in the left axilla treated for many years as hidradenitis suppurativa in her home country who was later found to have scrofuloderma. The diagnosis was based on a positive *Mycobacterium tuberculosis* polymerase chain reaction from an ultrasound-guided aspiration. Further investigation excluded pulmonary tuberculosis. In cases with an atypical presentation of hidradenitis suppurativa, imaging, along with histological and microbiologic examination are warranted to exclude scrofuloderma.

## Introduction

Pulmonary tuberculosis (TB) is a prevalent disease worldwide, representing a serious public health burden.^1^ Cutaneous TB, however, is relatively uncommon, comprising only 1%–2% of all extra-pulmonary manifestations of TB.^2–4^ It can be caused by *M. tuberculosis, Mycobacterium bovis* and the Bacillus Calmette–Guérin (BCG) vaccination.^5^ Cutaneous findings associated to TB include infectious lesions acquired exogenously by direct inoculation or endogenously by spread of a pre-existing internal TB infection, tuberculids, and reactions to the BCG vaccine.^3,5,6^

One form of endogenous TB is scrofuloderma, where the skin represents contiguous involvement from a focus of TB in an underlying lymph node or bone. This form is more commonly seen in children.^3,7^ Treatment for scrofuloderma is the same as for pulmonary TB, and clinical improvement is generally noted between the fourth and sixth weeks of treatment.^2,4^

We present a case of scrofuloderma which was initially misdiagnosed as hidradenitis suppurativa (HS).

## Case report

A 46-year-old female refugee from the Dominican Republic with a history of hypertension presented at our outpatient clinic for management of left axillary HS, previously diagnosed 4 years ago in her country. Her HS nodules were confined to her left axilla and first presented four years prior. She denied ever having lesions in other folds. She had previously received courses of antibiotics in her country with limited improvement.

She was initially treated with intralesional triamcinolone, topical clindamycine, and oral doxycycline. At her 2-month follow-up, the nodules had progressed, and she now presented two subcutaneous fluctuant masses underlying the HS nodules.

### Physical examination

On examination, three tender erythematous nodules with friable granulation tissue and sinus tracts were visible in the left axilla. On both the superior-lateral quadrant of the left breast and pectoral region, two large subcutaneous fluctuant masses were palpated. A solitary left cervical adenopathy was also palpated. No double comedones or HS nodules were visible in her axilla or other folds.

### Workup

Basic laboratory workup was unremarkable. The patient was referred to radiology for a surface ultrasound of the axilla, which demonstrated three heterogenous fluid collections in the left deltopectoral groove (5 × 2 × 3 cm), left subclavian space (1.8 × 1.6 cm and 2.8 × 2.4 cm), and supero-lateral quadrant of left breast (4.5 × 3.4 × 3.7 cm), all suggestive of suppurative adenitis. At this time, an ultrasound-guided aspiration of the fluid was conducted, which was positive for *M. tuberculosis* by polymerase chain reaction (PCR). Additional mycobacterial, bacterial, and fungal cultures of the fluid were negative. Chest X-ray and cervicothoracic computed tomography (CT)-scan showed normal pulmonary parenchyma and were negative for extra-axillary abnormalities. Cultures of sputum for *M. tuberculosis* were negative. She was not tested for HIV antibodies.

### Treatment

A diagnosis of scrofuloderma of the left axilla without pulmonary involvement was made. Infectious diseases were consulted, and the patient was treated with a combination of isoniazid, rifampicin, pyrazinamide, ethambutol, and vitamin B6. She was asked to self-isolate until the results of her sputum cultures were confirmed negative.

After 6 months of treatment, the patient demonstrated a near-complete resolution of the adenitis on follow-up CT-scan.

## Discussion

Scrofuloderma is a form of cutaneous TB arising from endogenous spread of the disease, by contiguous extension of the infection to the overlying skin from an infected deep structure like lymph nodes, bones, joints, or the epididymis.^4,5,8^ Scrofuloderma is often associated with pulmonary TB, particularly active pulmonary disease.^4,5^

The lesions of scrofuloderma start as mobile subcutaneous nodules which eventually attach to the overlying skin where cutaneous abscesses or draining sinus tracts can then form. Histology of true cutaneous TB reveals tuberculoid granulomas (epithelioid histiocytes and Langhans-type giant cells with a variable degree of central caseation necrosis and a peripheral rim of numerous lymphocytes). Acid-fast bacilli can be seen in biopsy material of the skin with Fite stain and on direct examination of abscess exudate.^5,6,8,9^

Cutaneous TB is treated with the same regimen as pulmonary TB. It consists of a prolonged multidrug therapy, most frequently involving rifampicin, isoniazid, pyrazinamide, and ethambutol for 8 weeks, followed by a maintenance phase of 16 weeks.^3,4,6^ Clinical improvement of the skin lesions is expected between the fourth and sixth week of treatment.^2^

**Figure 1.**
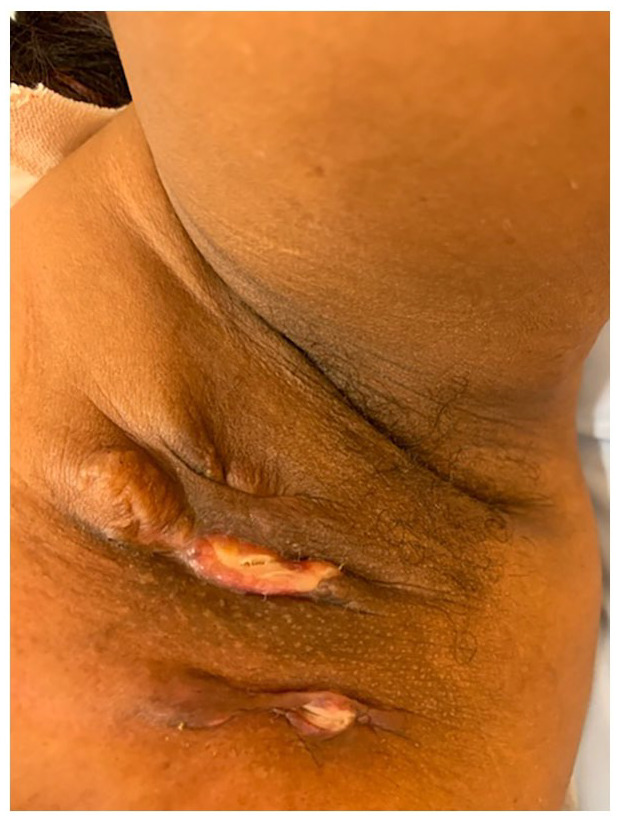
Left axilla: nodules with friable granulation tissue and sinus tracts, no double comedones.

The differential diagnosis of scrofuloderma includes HS, syphilitic gummas, paracoccidioidomycosis, coccidioidomycosis, sporotrichosis, actinomycosis, nocardiosis, and lymphogranuloma venereum.^5,9^ A few cases of scrofuloderma masquerading as HS have been described.^10,11^ In this case, initial misdiagnosis led to delay of treatment of her condition. It is important to maintain a healthy degree of suspicion for TB in cases of atypical HS.^7,11^ The late age of onset of the disease, its unilateral distribution, lack of involvement of other skin folds, and the presence of fluctuant subcutaneous masses prompted further investigation for this patient (Figure 1). It is especially vital to review an initial diagnosis of HS, as refractory HS is often treated with tumor necrosis factor (TNF) alpha inhibitors, which can potentiate existing TB.^9,10^
